# *SOCS3* Methylation Partially Mediated the Association of Exposure to Triclosan but Not Triclocarban with Type 2 Diabetes Mellitus: A Case-Control Study

**DOI:** 10.3390/ijms252212113

**Published:** 2024-11-11

**Authors:** Qian Gao, Changsheng Huan, Zexin Jia, Qingqing Cao, Pengcheng Yuan, Xin Li, Chongjian Wang, Zhenxing Mao, Wenqian Huo

**Affiliations:** 1 Department of Occupational and Environmental Health, College of Public Health, Zhengzhou University, 100 Kexue Avenue, Zhengzhou 450001, China; gq18730099825@163.com (Q.G.);; 2 Department of Epidemiology and Biostatistics, College of Public Health, Zhengzhou University, Zhengzhou 450001, China

**Keywords:** triclosan, triclocarban, *SOCS3* methylation, T2DM, glucose metabolism-related indicators

## Abstract

This study aimed to evaluate the association of TCs (triclosan (TCS) and triclocarban) exposure with T2DM and glucose metabolism-related indicators and the mediating effect of *SOCS3* methylation on their associations. A total of 956 participants (330 T2DM and 626 controls) were included in this case-control study. Logistic regression and generalized linear models were used to assess the effect of TCs on T2DM and glucose metabolism-related indicators. The dose–response relationship between TCs and T2DM was analyzed by restricted cubic spline. Finally, after evaluating the association between TCs and *SOCS3* methylation levels, the mediating effect of *SOCS3* methylation on the TC−associated T2DM was estimated. Each 1-unit increase in TCS levels was associated with a 13.2% increase in the risk of T2DM (OR = 1.132, 95% CI: 1.062, 1.207). A linear dose–response relationship was found between TCS and T2DM. TCS was negatively associated with *Chr17:76356190* methylation. Moreover, mediation analysis revealed that *Chr17:76356190* methylation mediated 14.54% of the relationship between TCS exposure and T2DM. Exposure to TCS was associated with a higher prevalence of T2DM. *SOCS3* methylation partially mediated the association of TCS with T2DM. Our findings may provide new insights into the treatment of T2DM, and the study of the biological mechanisms of T2DM.

## 1. Introduction

Since 1990, diabetes has become one of the global public health problems [1]. According to the latest International Diabetes Federation (IDF) Diabetes Atlas, the prevalence of diabetes among adults aged 20–79 will be as high as 12.2% in 2045 [2]. Among people with diabetes, 90 out of 100 patients are type 2 diabetes mellitus (T2DM) patients [3]. T2DM is typically characterized by persistently elevated blood glucose levels and insulin resistance [4]. It causes serious complications such as cognitive impairment, diabetic retinopathy, and diabetic nephropathy [5,6,7]. Additionally, diabetes during pregnancy can adversely affect the normal growth and development of the fetal brain [8]. However, due to the complexity of the pathogenesis of T2DM, the underlying cause of the disease has not yet been identified.

Triclosan (TCS) and triclocarban (TCC), as spectral antimicrobial agents, are used in the manufacturing of cosmetics and personal care products such as soaps, hand sanitizers, and toothpaste, as well as for sterilization in hospital environments [9,10]. In addition, TCS and TCC are components of plastic additives and are often found in plastic products like toys [11]. Therefore, humans are widely exposed to TCS and TCC through oral ingestion and dermal absorption [12,13]. An evidence-based review has demonstrated that environmental endocrine disruptors (EDCs) impair insulin secretion and its sensitivity [14]. Numerous studies have found that several common kinds of EDCs such as bisphenol A, parabens, and phthalates are associated with the development of T2DM [15,16]. However, few studies have explored the association of TCS and TCC, which are also EDCs, with the risk of T2DM [17].

As far as we know, only epidemiologic studies among the European and American populations were conducted to explore the association of TCS and TCC exposure with T2DM [18,19]. A sex-stratified study in the American population found that TCC was significantly and positively associated with T2DM in women, while no association between TCS and T2DM was observed [19]. Several studies have focused on pregnant women to estimate the association of TCS and TCC exposure with gestational diabetes mellitus (GDM); however, inconsistent results have been observed in these studies [20,21]. For example, a clinic-based case-control study did not find an association between TCS and GDM through a logistic model and a Bayesian kernel machine regression (BKMR) model [22]. The Shanghai Birth Cohort, the first Chinese study to examine the association between TCS exposure and GDM, reported that high levels of TCS were associated with an increased risk of GDM [23]. Different study populations and varying exposure levels of TCS and TCC may cause these inconsistent results [24]. Up to now, the effect of TCS and TCC exposure on T2DM has not been studied in the general adult Chinese population. Hence, it is crucial to clarify the exposure of TCS and TCC in rural Chinese populations and whether their exposure is associated with T2DM.

An animal study on pregnant mice found that TCS-treated mice had increased fasting blood glucose (FBG), insulin (INS), and homeostasis model assessment 2 of insulin resistance (HOMA2-IR) levels, and decreased homeostasis model assessment 2 of β cell function (HOMA2-β) [25]. However, the precise mechanism underlying the development of T2DM induced by exposure to TCS and TCC still necessitates further investigation. Several studies have revealed that DNA methylation may regulate the expression of the insulin gene promoter and inhibit gene transcription, which in turn leads to impaired insulin secretion and induces the development of T2DM [26,27]. The suppressor of cytokine signaling 3 (*SOCS3*), which belongs to the *SOCS* family of proteins, is a negative regulator of the activation of Janus kinase (JAK) and signal transducer and activator of transcription (STAT) [28]. Studies have shown that *SOCS3* is a vital risk factor for T2DM as it can inhibit insulin signaling and JAK/STAT signal channel conduction, lead to insulin resistance and higher levels of blood glucose [29,30,31]. An epigenomic-wide association study showed that hypermethylation of *SOCS3* was negatively associated with the risk of T2DM [32]. Our previous study also found that a higher DNA methylation level of *SOCS3* was significantly associated with a reduced risk of T2DM in a rural Chinese population [33]. Moreover, there is evidence showing TCS exposure can alter the expression of *SCOS* family. One study on chicken embryos found that embryonic injection of TCS significantly increased the expression level of embryonic *SOCS1* (which belongs to the same protein family as *SOCS3*) [34]. Another study, using human embryonic stem cells screened for differential gene expression of DNA methylation, identified that TCS exposure was significantly associated with upregulation of *SOCS3* expression [35]. However, the exact mechanism on how TCS exposure affect the expression of *SOCS3* remains unclear. One recent zebrafish study using reduced representative bisulfite sequencing (RRBS) technology has found that exposure to TCS altered the DNA methylation in zebrafish embryos and led to changes in the transcription of genes for important organ development [36], indicating the capability of TCS to alter gene expression by affecting its levels of DNA methylation. Therefore, we speculate that exposure to TCS may affect the expression of *SOCS3* through its DNA methylation. Abnormal gene methylation can affect the expression of certain genes in our body, triggering the occurrence of related diseases such as T2DM and lipid metabolism-related diseases [37,38]. Thus, we hypothesize that exposure to TCS and TCC may alter the methylation level of *SOCS3*, thereby inducing T2DM.

Therefore, taking the advantage of Henan Rural cohort study, we conducted a case-control study to investigate the above two hypotheses. First, the effects of TCS and TCC exposure on T2DM and glucose metabolism-related indicators were assessed. Then, we explored the mediating role of *SOCS3* methylation on the association between TCS and T2DM and glucose metabolism-related indicators and the potential mechanisms that may be involved.

## 2. Results

### 2.1. Basic Characteristics

Table 1 demonstrates the basic characteristics of the participants by T2DM. The age distribution of the participants was mostly 54–65. About 43.41% of the participants were men (*n* = 415). Compared to the control group, T2DM patients had less average monthly income, more current alcohol drinkers, a larger proportion of people with a family history of diabetes mellitus, and significantly higher levels of BMI, PP, and TG (all *p*-value < 0.05).

### 2.2. Distributions of Urinary TCS and TCC

As shown in Table S1, the detection rates (DRs) of TCS and TCC were 59.46% and 21.07%, respectively. The median concentration of TCS was 0.056 ng/mL. TCS and TCC presented a weak correlation (Spearman’s correlation coefficient = 0.203, *p* < 0.001).

In addition, the T2DM group had significantly higher concentrations of TCS and lower levels of *SOCS3* methylation than the control group (*p*-value < 0.05) (Table 1).

### 2.3. Association of TCS with T2DM and Glucose Metabolism-Related Indicators

Table 2 reveals the association of Ln-TCScrea with T2DM and glucose metabolism-related indicators. After adjusting for all confounders, Ln-TCScrea was associated with an increased risk of T2DM (OR = 1.132, 95% CI: 1.062, 1.207), which is comparable to epidemiological observations of increased risk of GDM by TCS exposure previously reported in pregnant women’s populations in the United States and Shanghai, China (Table S2) [21,23]. At the same time, TCS exposure significantly affected human blood glucose levels and increased insulin resistance, with significant increases in the levels of FBG (β = 0.135, 95% CI: 0.073, 0.196), INS (β = 0.157, 95% CI: 0.013, 0.302), HbA1c (β = 0.067, 95% CI: 0.028, 0.106), and HOMA2-IR (β = 0.015 95% CI: 0.004, 0.025). However, Ln-TCScrea was significantly associated with decreased HOMA2-β (β = −0.023, 95% CI: −0.038, −0.008), and the pancreatic β-cell function of participants was significantly reduced.

After trichotomizing the Ln-TCScrea concentration, except for INS levels, high levels of Ln-TCScrea were more significantly associated with T2DM and glucose metabolism-related indicators (*p* for trend < 0.05).

### 2.4. Dose–Response Relationship of TCS with T2DM and Glucose Metabolism-Related Indicators

**Figure 1:**
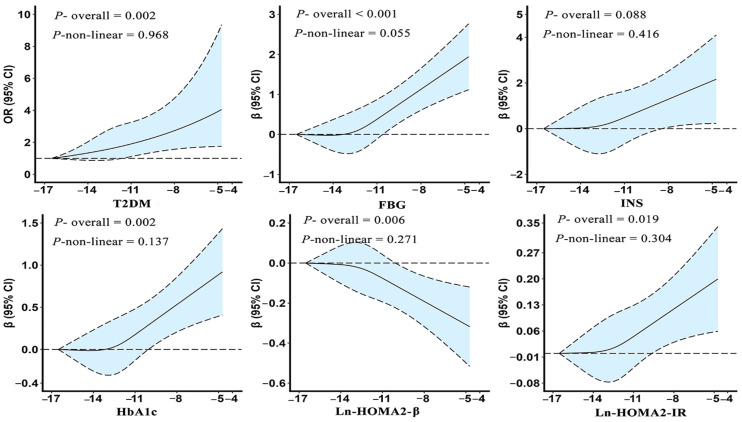
Dose–response relationship of Ln-TCScrea with T2DM and glucose metabolism-related indicators.

We further explored the dose–response relationship of Ln-TCScrea with T2DM and glucose metabolism-related indicators (Figure 1). The results show that Ln-TCScrea showed a significant linear relationship with the risk of T2DM and some glucose metabolism-related indicators (FBG, HbA1c, HOMA2-β, and HOMA2-IR) (*p* overall < 0.05, *p* non-linear > 0.05). That is, as the concentration of Ln-TCScrea increased, the risk of T2DM increased, and the glucose metabolism-related indicators increased or decreased (only HOMA2-β) in the monotonic change pattern.

### 2.5. Association of SOCS3 Methylation Levels with T2DM and TCS

The relationship between 93 CpG sites and four genomic regions and T2DM is shown in Tables S3 and S4. Hypermethylation of *SOCS3* was significantly associated with the reduced risk of T2DM. In particular, the methylation levels of *Chr17:76356190* and *Chr17:76356199* (*p* < 0.05/93) were negatively associated with T2DM. However, no association of genomic regions with T2DM was observed.

Table S5 shows the relationship between Ln-TCScrea and methylation levels of *Chr17:76356190* and *Chr17:76356199*. After adjusting for all covariates, it was found that the methylation level of *Chr17:76356190* was significantly negatively related to Ln-TCScrea (β = −0.025, 95% CI: −0.040, −0.009), and that exposure to TCS significantly reduced the methylation levels of *SOCS3*.

### 2.6. Mediating Effects of SOCS3 Methylation

**Figure 2:**
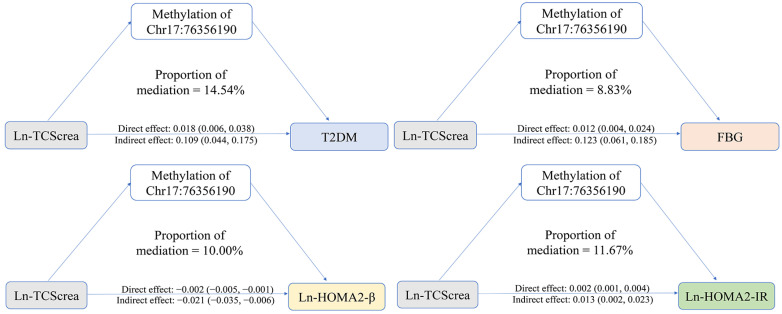
Mediating role of the methylation level of *Chr17:76356190* or *Chr17:76356199* between the association of Ln-TCScrea with T2DM and its glucose metabolism-related indicators.

Figure 2 and Table S6 describe the mediating effects of Chr17:76356190 and Chr17:76356199 methylation levels on the association of Ln-TCScrea with T2DM and glucose metabolism-related indicators. After adjusting for potential covariates, when the methylation levels of *Chr17:76356190* or *Chr17:76356199* were analyzed as mediators, the total effects of Ln-TCScrea on T2DM and all glucose metabolism-related indicators were significant. Next, the mediating effects of *Chr17:76356190* or *Chr17:76356199* methylation levels were examined. It was found that, to a certain extent, *Chr17:76356190* methylation levels mediated the association of Ln-TCScrea with T2DM and glucose metabolism-related indicators (FBG, HOMA2-β, and HOMA2-IR). Their indirect and direct effects were significant, with the proportions partially mediated being 14.54%, 8.83%, 10.00%, and 11.67%, respectively. However, there was no mediating effect of the methylation level of *Chr17:76356190* between the association of Ln-TCScrea with INS and HbA1c. Moreover, we also did not find any mediating effects of *Chr17:76356199* methylation levels between the associations of Ln-TCScrea with T2DM and glucose metabolism-related indicators.

### 2.7. Subgroup Analysis

The results of the subgroup analysis are shown in Table S7. No significant differences between categories of age, gender, education level, average monthly income, physical activity, smoking status, alcohol status, or BMI were observed in the association of Ln-TCScrea with T2DM, indicating that these factors have less impact on the effect of TCS exposure on T2DM.

### 2.8. Sensitivity Analysis

Considering the effect of performing diabetes treatment, we performed the sensitivity analysis after excluding patients who had received diabetes treatment within the last two weeks. The results showed that Ln-TCScrea maintained only a positive association with FBG, but the association with T2DM and other glucose metabolism-related indicators disappeared (Table S8).

## 3. Discussion

In this case-control study, we focused on the association of TCs (TCS and TCC) with T2DM and glucose metabolism-related indicators and the mediating effect of *SOCS3* methylation levels between their associations. Our findings showed that the rural Chinese populations are widely exposed to TCS. However, the DR and concentration of TCC were low. After adjusting for all confounders, the analysis revealed that TCS significantly increased the risk of T2DM. Meanwhile, TCS was significantly and positively associated with FBG, INS, and HbA1c levels and also disrupted glucose homeostasis. In addition, *Chr17:7635190* methylation levels significantly mediated the association between TCS and T2DM and glucose metabolism-related indicators (FBG, HOMA2-β, and HOMA2-IR).

Toxicologic and epidemiologic studies have investigated the association between TCS and TCC exposure and T2DM, but their results have been inconsistent. An animal experiment on *Xenopus tropicalis* found that exposure to TCS increased blood glucose levels, interfered with the activation of insulin receptors and insulin receptor substrates, and inhibited insulin signaling [39]. In addition, an experiment reported that TCC could affect hepatic glycolytic processes and cause abnormalities in hepatic glucose metabolism in male mice [40]. Animal studies revealed the adverse effects of TCS and TCC on glucose and insulin signaling; these are consistent with our significant results for TCS exposure with glucose metabolism-related indicators. For epidemiological studies, only three studies have estimated the association of TCS or TCC with T2DM, and they mainly focused on the American and European populations. One community-based, multiethnic longitudinal cohort study (SWAN cohort) did not find an association between TCS and incident diabetes in mid-life women [18]. However, another study using NHANES 2005–2014 data observed a significant negative association between TCS exposure and T2DM in American adults [41]. The other study using NHANES 2013–2014 data found a significant relationship between TCC but not TCS with T2DM among American adult women and no significant results among men [19]. More studies have focused on the association of TCS and TCC with GDM among pregnant women. Consistent with our findings, some TCS-GDM association studies have shown that pregnant women with high levels of TCS are more likely to have GDM [21,23]. For instance, a multiethnic longitudinal cohort study found that the third quartile of TCS was significantly and positively associated with GDM [21]. However, some investigations have unveiled that exposure to TCS was significantly related to the reduced risk of GDM [20,42]. One study showed that the antimicrobial effect of TCS could reduce systemic inflammation, thereby attenuating insulin resistance and providing protection against diabetes [41]. The possible reasons for the inconsistent results of the studies are different levels of exposure to TCS, different populations selected for the studies, or different criteria for the determination of diabetes [43]. Moreover, when performing sensitivity analyses, we excluded patients who had been treated for diabetes within the last two weeks, because treatment may improve individual endocrine levels, with which TCS is strongly correlated [44,45,46]. Our results show that, after excluding participants who had diabetes treatment within the last two weeks, the association between TCS and FBG remained significant. However, as the sample size decreased by deleting some participants, the associations of TCS with T2DM and the other four glucose metabolism indicators were no longer significant, but still maintained the direction of association consistent with the main results.

In addition, the development of T2DM is often inseparable from the mixed effects of environmental factors and genetic effects [47]. Numerous studies have also reported that the *SOCS3* is significantly associated with T2DM [29,48]. For example, one animal experiment on mice with muscle-specific overexpression of *SOCS3* found that increased *SOCS3* may mediate elevated insulin levels in obese mice [49]. The increased expression of *SOCS3* may induce the development of insulin resistance by inhibiting the JAK/STAT3 signaling pathway [50]. However, the expression of *SOCS3*, in addition to being affected by certain cytokines, is also inhibited by hypermethylation of *SOCS3* [51,52,53]. A nested case-control study using data from the London Life Sciences Prospective Population (LOLIPOP) study found that higher methylation levels of *SOCS3* were associated with a lower risk of T2DM in Asians [54]. Another study also showed that hypomethylation levels of *SOCS3* were highly associated with T2DM in HIV patients [55]. In the current study, we also reached the consistent result that *SOCS3* methylation (*Chr17:7635190* and *Chr17:7635199*) was significantly negatively associated with T2DM.

As far as we know, this is the first study to investigate the association of TCS exposure and *SOCS3* methylation. At present, although direct evidence about the effect of TCS on *SOCS3* is lacking, the following explanation may be plausible. First of all, TCS, as one kind of EDC, could act as an estrogen-like substance that can potentiate estrogenic effects at certain concentrations [56]. Estrogen is important for weight maintenance and metabolic health [18]. Estradiol in the normal physiological range favors insulin sensitivity, but out of the physiological range has an adverse effect [57]. Several studies have demonstrated that bisphenols (which belong to the same family of phenolics as TCS) would activate the JAK2/STAT3 pathway and regulate *SOCS3* expression by binding to estrogen receptors and exerting estrogen-like effects [50,58,59]. Therefore, TCS, which is also a phenolic, may also be able to regulate *SCOS3* expression by binding to estrogen receptors. Secondly, TCS was found to be genetically hepatotoxic, inhibiting DNA methylation transferase (DNMT1) activity in human hepatocytes (HepG2), thereby reducing DNA methylation levels [60]. Our results showed that the level of *SOCS3* methylation decreased with increasing TCS concentration. These indicated that TCS exposure may increase *SOCS3* expression by decreasing *SOCS3* methylation.

Going further, we performed mediation analyses to determine whether *SOCS3* methylation mediated the association of TCS exposure with T2DM. The results revealed that hypomethylation of *SOCS3* partially mediated the effect of exposure to TCS on the risk of T2DM, in which we first identified the mediating role of *Chr17:7635190* methylation levels in the association between TCS and T2DM. As mentioned above, we propose that TCS exposure will lead to low methylation levels of *SOCS3*, which causes high expression of *SOCS3*, and then increases the risk of T2DM. More specifically, as illustrated in the graphical abstract, a plausible explanation is that TCS exposure causes hypomethylation of *SOCS3*, and low methylation levels of *SOCS3* lead to an increase in its protein translation, and the SOCS3 protein binds to phosphorylated JAK and its receptor, attenuating the activation of STAT3, blocking the JAK2/STAT3 pathway and thus preventing insulin from performing its normal physiological function [61,62,63,64,65,66]. However, the specific biological mechanism underlying the mediation effect of *SOCS3* methylation on the relationship between TCS and T2DM and glucose metabolism-related indicators remains unclear. More rigorously designed prospective studies and animal experiments are essential to deepen our understanding.

Several studies have demonstrated that EDCs can induce oxidative stress and inflammation, leading to elevated in vivo levels of inflammatory cytokines, which subsequently impact insulin signaling and contribute to insulin resistance and T2DM [67]. Interleukin -6 (IL-6), as a cytokine, has been identified as a potent inhibitor of STAT3 activation, which can inhibit insulin from performing its function by affecting the JAK/STAT3/SOCS3 signaling pathway [53,68]. However, no current studies have specifically explored the relationship between TCS, IL-6, *SOCS3,* and T2DM. Although our study suggests that *SOCS3* may affect T2DM induced by exposure to TCS, due to the lack of corresponding toxicological experiments and metabolomics studies, we are unable to conclusively state that TCS can prevent insulin signaling and ultimately induce the development of T2DM.by increasing the level of IL-6 and affecting the methylation of *SOCS3*. Future relevant experiments are needed to validate our hypothesis.

To our knowledge, this is the first epidemiological study to explore the mediating effect of *SOCS3* methylation levels in the association between TCS and T2DM and glucose metabolism-related indicators. Besides the association study, we also investigated the possible epigenetic mechanism of TCS exposure on T2DM. Different from most previous studies focusing on TCS exposure and diabetes, we not only included T2DM as the outcome but also glucose metabolism-related indicators (FBG, INS, HbA1c, HOMA2-β, and HOMA2-IR). These glucose metabolism-related indicators change before T2DM is diagnosed, so they act as biomarkers for early diagnosis of T2DM [69]. In the present study, we found the consistent change trends of these glucose metabolism-related indicators with T2DM, which further demonstrated the adverse effect of TCS exposure on the risk of T2DM.

Of course, our study has several limitations. Firstly, restricted by the low level of causal inference in the case-control design, this study could not provide evidence of a direct causal association between TCS exposure and increased risk of T2DM. Secondly, humans are often exposed to multiple kinds of EDCs at the same time [70], so our study could not exclude the effects of other pollutants. More comprehensive multi-pollutant mixing analyses will be needed in the future to assess the overall effect of EDCs on T2DM. Finally, we detected only one gene, *SOCS3*, in whole blood samples, which cannot fully expound the mechanism that triggers T2DM. We need to increase the detection of related genes to explore the specific mechanism in the future.

## 4. Methods and Materials

### 4.1. Study Population

**Figure 3:**
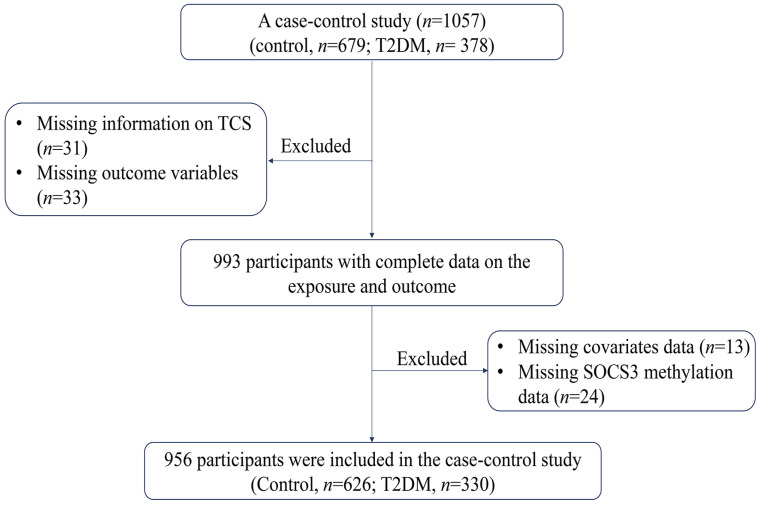
The flowchart of the inclusion and exclusion of participants. Abbreviations: T2DM, type 2 diabetes mellitus; TCS, triclosan.

Based on the Henan Rural cohort (Chinese Clinical Trial Registration Number: ChiCTR-OOC-15006699) [71], we selected the study population from the Suiping County baseline population with a sample size of 6670 and conducted this case-control study. Firstly, 378 patients with T2DM and 679 controls were randomly selected. As shown in Figure 3, a total of 64 participants were excluded due to missing data on TCS (*n* = 31) and glucose metabolism-related indicators (*n* = 33), respectively. Then, there were 13 participants with missing information on relevant covariates and 24 participants with missing *SOCS3* methylation information were excluded from the study. Ultimately, 956 participants were included in the study for analysis, including 330 T2DM patients and 626 controls.

This case-control study, following the principles of the Declaration of Helsinki, was approved by the Life Sciences Ethics Committee of Zhengzhou University (Code: [2015] MEC (S128)). In addition, all participants signed an informed consent form.

### 4.2. Laboratory Measurements

After participants had fasted for at least 8 h, blood and urine samples were collected. Serum and plasma were separated and stored in a refrigerator at −80 °C (the same storage conditions were used for urine samples), until it was necessary to use the samples for chemical analysis. Serum FBG and INS levels were measured by a Roche Cobas 501 (Basel, Switzerland) automatic biochemical analyzer and radioimmunoassay, respectively. High-performance liquid chromatography (HPLC) (Waters XEVO TQ-S system (Waters, Milford, MA, USA)) was used to determine the level of whole glycosylated hemoglobin (HbA1c). Finally, the glucose homeostasis parameters (HOMA2-β, HOMA2-IR) of the participants were calculated by HOMA calculator software (v2.2.3). In addition, the Roche Cobas 501 was also used to quantify urinary creatinine levels.

The determination of TCS and TCC concentrations in urine was divided into 3 steps. (1) Incubation: 1 mL of urine sample was taken into a polypropylene tube, and 0.3 mL of buffer and 50 μL of internal standard mixture (100 μg/L) were added sequentially, followed by shaking for 30 min and incubation for 6 h at 37 °C. (2) Extraction: After the incubation was completed, the sample was added with 0.5mL acetonitrile and 3 mL ethyl acetate, and sonicated and shaken for 30 min each before the supernatant was extracted. The above extraction operation was repeated twice. The supernatants from the two extractions were combined and blown to near dryness with nitrogen. (3) Sample preparation: Blow-dried samples were re-dissolved in 200 μL of 50% methanol-water, shaken for 2~3 min, filtered through 0.22 μm organic filter membrane, and then transferred to the injection bottle. Finally, TCS and TCC in urine were analyzed using the ultra-performance liquid chromatography-tandem mass spectrometry (UPLC-MS/MS) (Waters XEVO TQ-S) system.

The concentrations of TCS and TCC were quantified by UPLC-MS/MS. The chromatographic column was an ACQUITY UPLC BEH C18 column (2.1 mm × 100 mm, 1.7 μm, Waters, Ireland) at 40 °C. The injection volume was 5 μL, and the velocity was 0.3 mL/min. The mobile phases were pure water (solvent A) and methanol (solvent B). The gradient elution procedure is shown in Table S9. Electrospray ionization-negative (ESI-) mode was used to identify the target compounds. The quantification of the target compounds was carried out by the multi-reaction monitoring (MRM) mode, with a residence time of 2 ms, and the temperature of the ion source was 120 °C. The nitrogen flow rate was 900 L/h and the collision chamber pressure was 2.92 × 10^−3^ psi. Other mass spectrometry parameters are shown in Table S10.

Genomic DNA was extracted using the Whole Blood Genomic DNA Extraction Kit III (magnetic bead) (Bioteke Corporation, Beijing, China). The methylation levels of the four regions of the SOCS3 gene, containing 93 CpG sites, were detected (Table S3). Gel electrophoresis was employed to evaluate DNA purity, ensuring that the DNA samples were free from significant degradation and contamination. Primer design and gene sequencing procedures have been reported in previous articles [33,72].

After mixing the primers, the genomic DNA was amplified via multiplex polymerase chain reaction (PCR). Each PCR reaction system totaled 20 μL, which included 1×reaction butter, Mg2^+^ (2 mM), dNTP (0.2 mM), primers (0.1 μM each), template DNA (2.0 μL), and 1 U HotStarTaq polymerase. Genomic DNA was processed with the EZ DNA Methylation-Gold Kit (ZYMO, Irvine, CA, USA) and unmethylated C was converted to U. Then, specific tag sequences compatible with the Illumina (Illumina, San Diego, CA, USA) platform were introduced into the end of the library by PCR amplification using primers labelled with Index sequences. Each PCR reaction system also had a total volume of 20 μL, consisting of 1×reaction butter, dNTP (0.3 mM), F primer (0.25 μM), index primer (0.25 μM), template (1.0 μL), and 1 U of Q5TM DNA polymerase. Next, PCR amplification products were mixed in equal proportions and subjected to high-throughput sequencing on the Illumina Hiseq/Miseq platform. Finally, the methylation levels of *SOCS3* were evaluated using MethylTarget (Genesky, Shanghai, China).

Additionally, systolic blood pressure (SBP) and diastolic blood pressure (DBP) were measured using an electronic blood pressure monitor (Omron HEM-7071A, Kyoto, Japan), and three measurements were taken and averaged for analysis. Triglyceride (TG), and total cholesterol (TC) were determined using Roche Cobas 501 automatic biochemical analyzer.

### 4.3. Quality Control

In the quality control, three spiked samples were prepared at three different levels, five parallel samples were set up for each level and measured simultaneously three times in one day to calculate the intra-day coefficient of variation. Then, the samples were injected for three consecutive days to calculate the inter-day coefficient of variation. In addition, for every 12 samples, one blank and one spiked sample were tested to ensure that the results were reliable. Quantitative analyses were performed using a 9-point calibration curve with regression coefficients greater than 0.99 over the range of 0.1 ng/mL ~ 50 μg/L. The limit of detection (LOD) was defined as 3 times the signal-to-noise ratio. The LOD of TCS and TCC were 0.0293 and 0.066 ng/mL, respectively. Detailed information such as recoveries and coefficients of variation are shown in Table S11.

### 4.4. Definition of T2DM

Participants were considered to have T2DM if they had any of the following plasma glucose values: (1) FBG ≥ 7.0 mmol/L; (2) HbA1c ≥ 6.5%; and (3) self-reported history of T2DM and use of glucose-lowering medications in the past two weeks [73,74].

### 4.5. Definition of Covariates

The covariates consisted of four main components: basic demographic characteristics, lifestyle information, family history of diabetes mellitus information, and measurement indicators. They were obtained by trained investigators through structured questionnaires and specialized instruments.

Basic demographic characteristics included age, gender, education level (never attended school, primary school, junior secondary and above), marital status (married/cohabiting, widowed/single/divorced), average monthly income (CNY < 500, CNY 500~, CNY 1000~). A total of 5 items were included in the participant’s lifestyle, including smoking status (current, never/past), alcohol status (current, never/past), physical activity (low, moderate, high), high-fat diet (yes (>75 g/day), no), vegetable and fruit intake (yes (>500 g/day), no)). A participant was considered to have a family history of T2DM if one of his or her parents or siblings had been diagnosed with T2DM, and none otherwise. Participants who smoked at least one cigarette per day for more than six continuous months were defined as current smokers. Participants were considered current drinkers if they consumed alcohol at least 12 times each year. According to the guidelines of the International Physical Activity Questionnaire, short version (IPAQ-short), physical activity was categorized as low, medium, and high levels. Participants who consumed no less than 75 g of fat per day were defined as having a high-fat diet. Daily intake of vegetables and fruits not less than 500 g was defined as a high intake of vegetables and fruits.

Body mass index (BMI) was calculated as weight (kg)/height (m) squared. Pulse pressure (PP) = SBP − DBP. In addition, TG and TC were also included as covariates.

### 4.6. Statistical Analysis

The Kolmogorov–Smirnov test was used to check whether the variables conformed to normal distribution. Concentrations of urinary TCS and TCC below the LOD were replaced by LOD/2. TCC was not included in subsequent analyses because of its low DR (<50%). Urinary TCS concentrations were corrected for urinary creatinine and then natural logarithmically transformed (Ln-TCScrea) for its abnormal distribution. Mean (standard deviation, SD) or median (IQR) were used to describe the characteristics of continuous variables; in addition, the characteristics of categorical variables were expressed as numbers (percentages). To identify differences between case and control groups, the *t*-test was used to analyze normally distributed continuous variables, the Mann–Whitney U-test for skewed continuous variables, and the Chi-square test for categorical variables. Spearman correlation analysis was used to evaluate the correlation between TCS and TCC.

A one-way logistic regression model was used to assess the association of *SOCS3* methylation (including 93 CpG sites and 4 regions) with T2DM, and two-sided *p* < 0.05/93 or *p* < 0.05/4 indicated statistical significance. In addition, the relationship between TCS and *SOCS3* methylation levels was examined by linear regression model.

A logistic regression model was used to analyze the relationship between TCS and T2DM, and a generalized linear model was used to assess the relationship between TCS and glucose metabolism-related indicators. Three models were used in this study, including model 1 (crude model), model 2 (adjusted for age, gender, education level, marital status, average monthly income, smoking status, alcohol status, physical activity, vegetable and fruit intake, and high-fat diet, family history of T2DM), and model 3 (based on model 2 + BMI, PP, TC, TG). The dose–response relationship between TCS and target outcomes was further explored by restricted cubic spline. Next, mediation analysis was performed by SPSS PROCESS to assess the mediation effects of the methylation levels of *SOCS3* on the association of TCS with T2DM and glucose metabolism-related indicators, as well as the explainable proportions (bootstrap= 1000). Subgroup analysis was also conducted according to characteristics such as age, gender, and the potential interaction effects of pollutants with these characteristics were analyzed. Finally, participants who underwent diabetes treatment in the last two weeks were excluded from sensitivity analysis.

Data were analyzed using SPSS 21.0 and R 4.2.2. *p* < 0.05 was considered a statistically significant difference.

## 5. Conclusions

In summary, exposure to TCS significantly increased the risk of T2DM. In addition, *Chr17:76356190* methylation partially mediated the effects of TCS on T2DM and glucose metabolism-related indicators. Although our proposed biological mechanisms need to be validated by further prospective studies and animal experiments, our findings still lay a theoretical foundation and provide a research direction for the biological mechanisms that induce the development of T2DM.

## Figures and Tables

**Table 1:** Characteristics of the study population by T2DM.

Characteristics	Whole	Control	T2DM	*p*-Value ^a^
	(*n* = 956)	(*n* = 626)	(*n* = 330)	
Age, years, median (IQR)	61 (54, 65)	61 (54, 65)	61 (54, 65)	0.931
<55	325 (34.00)	211 (33.71)	114 (34.55)	
55–65	405 (42.36)	266 (42.49)	139 (42.12)	
>65	226 (23.64)	149 (23.80)	77 (23.33)	
Men, *n* (%)	415 (43.41)	275 (43.93)	140 (42.42)	0.655
Educational level, *n* (%)				0.990
never attended school	242 (25.31)	159 (25.40)	83 (25.15)	
primary school	294 (30.75)	193 (30.83)	101 (30.61)	
Junior, secondary, and above	420 (43.94)	274 (43.77)	146 (44.24)	
Marital status, *n* (%)				0.315
married/cohabiting	856 (89.54)	556 (88.82)	300 (90.91)	
widowed/single/divorced	100 (10.46)	70 (11.18)	30 (9.09)	
Average monthly income, *n* (%)				**0.011**
CNY <500	374 (39.12)	229 (36.58)	145 (43.94)	
CNY 500~	310 (32.43)	223 (35.62)	87 (26.36)	
CNY 1000~	272 (28.45)	174 (27.80)	98 (29.70)	
Smoking status, *n* (%)				0.063
current	191 (19.98)	136 (21.73)	55 (16.67)	
never/past	765 (80.02)	490 (78.27)	275 (83.33)	
Alcohol status, *n* (%)				**0.025**
current	148 (15.48)	85 (13.58)	63 (19.09)	
never/past	808 (84.52)	541 (86.42)	267 (80.91)	
Physical activity, *n* (%)				0.404
low	249 (26.05)	156 (24.92)	93 (28.18)	
moderate	427 (44.67)	279 (44.57)	148 (44.85)	
high	280 (29.28)	191 (30.51)	89 (26.97)	
High-fat diet (>75 g/day, *n* (%))	188 (19.67)	120 (19.17)	68 (20.61)	0.595
Vegetable and fruit intake (>500 g/day, *n* (%))	618 (64.64)	405 (64.70)	213 (64.55)	0.963
Family history of T2DM, *n* (%)	28 (2.93)	7 (1.12)	21 (6.36)	**<0.001**
BMI, kg/m^2^, mean ± SD	24.42 ± 3.65	23.32 ± 2.94	26.50 ± 3.94	**<0.001**
<18.5	19 (1.99)	14 (2.24)	5 (1.52)	
18.5–23.9	459 (48.01)	371 (59.27)	88 (26.67)	
>23.9	478 (50.00)	241 (38.49)	237 (71.83)	
PP (mm Hg), median (IQR)	47 (41, 56)	46 (40, 55)	49 (42, 58)	**0.001**
TC (mmol/L), median (IQR)	4.66 (4.11, 5.32)	4.63 (4.09, 5.23)	4.73 (4.19, 5.45)	0.052
TG (mmol/L), median (IQR)	1.61 (1.12, 2.42)	1.47 (1.02, 2.20)	1.88 (1.32, 2.92)	**<0.001**
Pollutants, median (IQR)				
TCS, (ng/mL)	0.056 (0.003, 0.236)	0.040 (0.003, 0.182)	0.107 (0.003, 0.334)	**<0.001**
TCC, (ng/mL)	<LOD	<LOD	<LOD	
SOCS-3 DNA methylation (%), mean ± SD				
Methylation of *Chr17:76356190*	1.05 ± 0.62	1.13 ± 0.66	0.88 ± 0.49	**<0.001**
Methylation of *Chr17:76356199*	0.95 ± 0.38	0.99 ± 0.40	0.86 ± 0.32	**<0.001**
Genomic region, mean ± SD				
*Chr17:76355106*_*Chr17:76355374*	1.61 ± 0.38	1.61 ± 0.38	1.62 ± 0.39	0.624
*Chr17:76356032*_*Chr17:76356279*	0.96 ± 0.16	0.96 ± 0.15	0.97 ± 0.16	0.607
*Chr17:76354901*_*Chr17:76355135*	31.28 ± 8.77	31.36 ± 9.04	31.15 ± 8.26	0.723
*Chr17:76354539*_*Chr17: 76354788*	76.66 ± 4.14	76.62 ± 4.20	76.72 ± 4.03	0.729

Abbreviation: SD, standard deviation; IQR, interquartile range; BMI, body mass index; PP, pulse pressure; TC, total cholesterol; TG, triglyceride; TCS, Triclosan; TCC, triclocarban; T2DM, type 2 diabetes mellitus. ^a^ Differences of continuous and categorical covariates conforming to normal distribution between control and T2DM were tested by *t*-test and Chi-square test, respectively. Continuous variables not conforming to normal distribution were tested by Mann–Whitney U-tests.

**Table 2:** Odds ratios/*β* (95% confidence intervals) of Ln-TCScrea in urine to T2DM and glucose metabolism-related indicators.

Outcome	*OR* (95%CI)	*β* (95%CI)				
	T2DM	FBG	INS	HbA1c	Ln-HOMA2-β	Ln-HOMA2-IR
**model 1**						
Continuous	**1.134 (1.073, 1.198)**	**0.159 (0.093, 0.225)**	**0.226 (0.072, 0.379)**	**0.081 (0.039, 0.122)**	**−0.027 (−0.042, −0.011)**	**0.021 (0.009, 0.032)**
T1	Reference	Reference	Reference	Reference	Reference	Reference
T2	1.352 (0.964, 1.898)	0.320 (−0.078, 0.717)	0.677 (−0.246, 1.600)	0.107 (−0.140, 0.354)	−0.042 (−0.133, 0.049)	0.061 (−0.008, 0.130)
T3	**1.982 (1.422,2.761)**	**0.770 (0.373, 1.168)**	0.815 (−0.108, 1.738)	**0.474 (0.227, 0.721)**	**−0.150 (−0.241, −0.059)**	**0.083 (0.014, 0.151)**
*p* for trend	**<0.001**	**<0.001**	0.083	**<0.001**	**0.001**	**0.018**
**model 2**						
Continuous	**1.138 (1.075, 1.205)**	**0.162 (0.097, 0.227)**	**0.223 (0.069, 0.377)**	**0.082 (0.042, 0.123)**	**−0.027 (−0.042, −0.012)**	**0.020 (0.009, 0.032)**
T1	Reference	Reference	Reference	Reference	Reference	Reference
T2	1.388 (0.976, 1.973)	0.350 (−0.043, 0.743)	0.699 (−0.228, 1.626)	0.138 (−0.106, 0.383)	−0.046 (−0.136, 0.044)	0.062 (−0.007, 0.131)
T3	**2.000 (1.416, 2.824)**	**0.768 (0.376, 1.160)**	0.799 (−0.126, 1.724)	**0.475 (0.231, 0.719)**	**−0.147 (−0.237, −0.057)**	**0.080 (0.011, 0.148)**
*p* for trend	**<0.001**	**<0.001**	0.091	**<0.001**	**0.001**	**0.023**
**model 3**						
Continuous	**1.132 (1.062, 1.207)**	**0.135 (0.073, 0.196)**	**0.157 (0.013, 0.302)**	**0.067 (0.028, 0.106)**	**−0.023 (−0.038, −0.008)**	**0.015 (0.004, 0.025)**
T1	Reference	Reference	Reference	Reference	Reference	Reference
T2	1.260 (0.853, 1.861)	0.253 (−0.119, 0.625)	0.442 (−0.425, 1.309)	0.082 (−0.150, 0.313)	−0.030 (−0.119, 0.058)	0.040 (−0.023, 0.103)
T3	**1.776 (1.207, 2.614)**	**0.573 (0.200, 0.945)**	0.289 (−0.580, 1.158)	**0.360 (0.128, 0.592)**	**−0.119 (−0.208, −0.031)**	0.036 (−0.027, 0.100)
*p* for trend	**0.003**	**0.003**	0.516	**0.002**	**0.008**	0.258

Abbreviation: TCS, Triclosan; T2DM, type 2 diabetes mellitus; FBG, fasting blood glucose; INS, insulin; HbA1c, glycosylated hemoglobin A1c; HOMA2-β, homeostasis model assessment 2 of β cell function; HOMA2-IR, homeostasis model assessment 2 of insulin resistance; model 1, crude model; model 2, adjusted age, gender, educational level, marital status, average monthly income, smoking status, alcohol status, physical activity, high-fat diet, vegetable and fruit intake, and family history of T2DM; model 3, model 2 + BMI, PP, TC, TG.

## Data Availability

The data that support the findings of this study are available upon reasonable request from the corresponding authors.

## Supplementary Materials

The following supporting information can be downloaded at: https://www.mdpi.com/article/10.3390/ijms252212113/s1.
