# Supplementary material for: SOCS3 Methylation Partially Mediated the Association of Exposure to Triclosan but Not Triclocarban with Type 2 Diabetes Mellitus: A Case-Control Study
Source: Int J Mol Sci. 2024 Nov 11;25(22):12113. doi: 10.3390/ijms252212113 (PMC11594987; doi:10.3390/ijms252212113)
Supplement: Supplementary file 1 [file ijms-25-12113-s001.zip › ijms-3291151-supplementary.pdf]

Supplementary Tables

Table S1 Distribution of urinary TCS and TCC concentrations (ng/mL) in participants.
Table S2 Association of triclosan and triclocarban exposure with type 2 diabetes mellitus/ gestational diabetes mellitus reported for different populations.
Table S3 CpG sites and *OR* (95%CI) for T2DM in univariate logistic regression model (*n*= 1057).
Table S4 Genomic region and *OR* (95%CI) for T2DM in univariate logistic regression model (*n*= 1057).
Table S5 Association between Ln-TCScrea and methylation level of Chr17:76356190 or Chr17:76356199.
Table S6 Mediation analysis of the association of Ln-TCScrea with T2DM and glucose metabolism-related indicators by methylation level of Chr17:76356190 or Chr17:76356199.
Table S7 The results of subgroup analysis.
Table S8 Sensitivity analysis (excluding patients who had diabetes treatment in the last two weeks) (*n*=784).
Table S9 HPLC-MS/MS optimized parameters for TCS and TCC.
Table S10 Liquid chromatography gradient elution program
Table S11 Spiked recoveries, coefficients of variation and detection limits of TCS and TCC.

Table S1 Distribution of urinary TCS and TCC concentrations (ng/mL) in participants.

**pollutants**	**DF （%）**	**Percentile**				
		**5th**	**25th**	**50th**	**75th**	**95th**
TCS	59.46	<LOD	<LOD	0.056	0.236	2.060
TCC	21.07	<LOD	<LOD	<LOD	<LOD	0.107

Abbreviation: DF, detection frequency; deviation; LOD, limits of detection; TCS, triclosan; TCC, triclocarban.

Table S2 Association of triclosan and triclocarban exposure with type 2 diabetes mellitus/ gestational diabetes mellitus reported for different populations.

Population	Sample size	TCS&T2DM/GDM	TCC&T2DM/GDM	Reference
Henan Rural Cohort Study	956	↑	–	This study
NHANES 2013-2014	900	–	↑(women)	Xie et al. (2020)
SWAN Multi-Pollutant Study (MPS)	1299	–	/	Li et al. (2021)
NHANES 2005-2014	8498	↓	/	Ward et al. (2020)
Cambridge Baby Growth Study （CBGS）	232	↓*	/	Fisher et al. (2018)
Pregnancy Environment and Lifestyle Study (PETALS)	111	↑*	/	Zhu et al. (2022)
Shanghai Birth Cohort Study	620	↑*	/	Ouyang et al. (2018)

Abbreviation: TCS, triclosan; TCC, triclocarban; T2DM, type 2 diabetes mellitus; GDM, gestational diabetes mellitus.

↑: positive association; ↓: negative association; –: no association; /: not studied; *: TCS&GDM.

Table S3 CpG sites and *OR* (95%CI) for T2DM in univariate logistic regression model.

CpG site	Position	Distance2TSS	*OR* (95%CI)	*P*-value
Chr17:76355136	31	1024	1.004(0.970,1.038)	0.835
Chr17:76355146	41	1014	1.076(0.935,1.239)	0.306
Chr17:76355149	44	1011	1.039(0.942,1.146)	0.442
Chr17:76355152	47	1008	1.001(0.922,1.087)	0.985
Chr17:76355179	74	981	1.004(0.949,1.061)	0.898
Chr17:76355183	78	977	1.116(0.930,1.339)	0.238
Chr17:76355192	87	968	1.367(1.071,1.745)	0.012
Chr17:76355202	97	958	0.925(0.734,1.166)	0.508
Chr17:76355204	99	956	1.213(0.933,1.578)	0.148
Chr17:76355207	102	953	1.105(0.943,1.295)	0.218
Chr17:76355210	105	950	0.982(0.807,1.194)	0.853
Chr17:76355225	120	935	1.026(0.809,1.302)	0.831
Chr17:76355227	122	933	1.067(0.836,1.360)	0.603
Chr17:76355234	129	926	1.000(0.833,1.200)	0.998
Chr17:76355241	136	919	1.045(0.784,1.393)	0.764
Chr17:76355245	140	915	1.187(0.974,1.446)	0.090
Chr17:76355251	146	909	1.243(0.978,1.580)	0.075
Chr17:76355258	153	902	1.087(0.868,1.361)	0.468
Chr17:76355268	163	892	0.954(0.782,1.164)	0.643
Chr17:76355280	175	880	0.901(0.745,1.089)	0.279
Chr17:76355282	177	878	0.731(0.568,0.941)	0.015
Chr17:76355286	181	874	0.833(0.659,1.053)	0.126
Chr17:76355288	183	872	0.997(0.772,1.287)	0.979
Chr17:76355299	194	861	0.982(0.784,1.230)	0.874
Chr17:76355312	207	848	0.912(0.694,1.198)	0.506
Chr17:76355314	209	846	0.953(0.730,1.244)	0.724
Chr17:76355318	213	842	1.238(0.933,1.641)	0.138
Chr17:76355323	218	837	1.100(0.933,1.297)	0.258
Chr17:76355328	223	832	0.942(0.681,1.303)	0.718
Chr17:76355343	238	817	1.096(0.833,1.441)	0.513
Chr17:76355346	241	814	1.021(0.794,1.314)	0.870
Chr17:76355350	245	810	1.396(1.004,1.941)	0.047
Chr17:76356054	23	106	0.998(0.780,1.276)	0.985
Chr17:76356056	25	104	1.264(0.872,1.831)	0.216
Chr17:76356067	36	93	1.186(0.960,1.466)	0.114
Chr17:76356069	38	91	1.180(0.860,1.620)	0.305
Chr17:76356072	41	88	1.216(0.851,1.738)	0.282
Chr17:76356084	53	76	1.013(0.863,1.189)	0.876
Chr17:76356088	57	72	1.177(0.816,1.700)	0.383
Chr17:76356092	61	68	1.162(0.807,1.675)	0.420
Chr17:76356099	68	61	0.990(0.667,1.468)	0.959
Chr17:76356109	78	51	1.042(0.744,1.459)	0.811

Chr17:76356113	82	47	1.028(0.698,1.515)	0.889
Chr17:76356119	88	41	1.421(1.035,1.952)	0.030
Chr17:76356124	93	36	1.227(0.886,1.699)	0.219
Chr17:76356133	102	27	0.934(0.636,1.372)	0.727
Chr17:76356152	121	8	1.179(0.813,1.711)	0.385
Chr17:76356158	127	2	1.194(0.839,1.699)	0.325
Chr17:76356161	130	-1	1.289(0.959,1.733)	0.093
Chr17:76356163	132	-3	1.057(0.738,1.514)	0.762
Chr17:76356170	139	-10	0.957(0.728,1.259)	0.755
Chr17:76356172	141	-12	1.012(0.687,1.491)	0.952
Chr17:76356176	145	-16	1.537(1.125,2.101)	0.007
Chr17:76356178	147	-18	1.088(0.822,1.441)	0.555
Chr17:76356190	159	-30	**0.521(0.412,0.660)**	**<0.001**
Chr17:76356194	163	-34	0.911(0.688,1.206)	0.513
Chr17:76356197	166	-37	0.867(0.640,1.174)	0.356
Chr17:76356199	168	-39	**0.435(0.305,0.622)**	**<0.001**
Chr17:76356204	173	-44	1.337(0.913,1.960)	0.136
Chr17:76356211	180	-51	1.214(0.908,1.623)	0.190
Chr17:76356226	195	-66	0.813(0.579,1.143)	0.234
Chr17:76356228	197	-68	0.941(0.681,1.302)	0.715
Chr17:76356232	201	-72	0.893(0.628,1.269)	0.527
Chr17:76354927	27	1233	0.999(0.985,1.014)	0.943
Chr17:76354934	34	1226	1.005(0.992,1.018)	0.473
Chr17:76354947	47	1213	1.002(0.989,1.014)	0.778
Chr17:76354955	55	1205	1.003(0.991,1.016)	0.614
Chr17:76354963	63	1197	0.998(0.983,1.013)	0.785
Chr17:76354965	65	1195	1.001(0.987,1.016)	0.866
Chr17:76354981	81	1179	0.990(0.964,1.017)	0.480
Chr17:76354984	84	1176	1.001(0.987,1.015)	0.862
Chr17:76354990	90	1170	0.998(0.972,1.025)	0.874
Chr17:76355009	109	1151	1.000(0.986,1.013)	0.969
Chr17:76355014	114	1146	1.003(0.990,1.015)	0.692
Chr17:76355017	117	1143	0.999(0.984,1.015)	0.906
Chr17:76355020	120	1140	1.000(0.985,1.015)	0.996
Chr17:76355029	129	1131	1.000(0.985,1.015)	0.990
Chr17:76355044	144	1116	1.000(0.987,1.013)	0.981
Chr17:76355061	161	1099	1.001(0.990,1.013)	0.812
Chr17:76355068	168	1092	0.998(0.986,1.011)	0.811
Chr17:76355089	189	1071	0.999(0.987,1.012)	0.918
Chr17:76355115	215	1045	0.998(0.985,1.010)	0.711
Chr17:76354582	44	1578	1.025(1.002,1.048)	0.032
Chr17:76354588	50	1572	1.018(0.989,1.047)	0.220
Chr17:76354596	58	1564	1.048(1.007,1.092)	0.021
Chr17:76354621	83	1539	1.000(0.982,1.018)	0.992

Chr17:76354638	100	1522	1.022(0.995,1.049)	0.111
Chr17:76354663	125	1497	1.013(0.985,1.041)	0.364
Chr17:76354669	131	1491	0.998(0.977,1.020)	0.869
Chr17:76354724	186	1436	1.002(0.984,1.021)	0.839
Chr17:7635732	194	1428	1.013(0.990,1.036)	0.277
Chr17:76354741	203	1419	1.007(0.992,1.024)	0.356
Chr17:76354763	225	1397	1.001(0.982,1.019)	0.944

Each tested CpG site was named as its genomic position.

Chr: The chromosome on which the fragment is located; Genomic Position: The position of the fragment on the reference genome; Position: The position of the CpG site on the target genomic region; Distance to TSS: The distance of the site on the reference genome relative to the transcription start site (TSS), and the minus sign indicates that the site is upstream of the TSS.

Abbreviations: CI, confidence interval; *OR*, odds ratio; T2DM, type 2 diabetes mellitus.

P <0.05 /93 is considered as statistically significant with two-tailed test.

Table S4 Genomic region and *OR* (95%CI) for T2DM in univariate logistic regression model.

Genomic region	Length	Distance to TSS	*OR* (95%CI)
Chr17:76355106_Chr17:76355374	269	786	1.083(0.769,1.524)
Chr17:76356032_Chr17:76356279	248	-119	1.117(0.499,2.500)
Chr17:76354901_Chr17:76355135	235	1025	0.999(0.984,1.014)
Chr17:76354539_Chr17: 76354788	250	1372	1.014(0.983,1.047)

Genomic region: Genomic region was named as the start site to the end site on the chromosome. Length: The product's size (bp); Distance to TSS: The distance of the site on the reference genome relative to the transcription start site (TSS), and the minus sign indicates that the site is upstream of the TSS.

Abbreviations: T2DM, type 2 diabetes mellitus.

$P < 0.05/4$ is considered as statistically significant with two-tailed test.

Table S5 Association between Ln-TCScrea and methylation level of Chr17:76356190 or Chr17:76356199.

**SOCS-3 DNA methylation**	***β* (95%CI)**		
	**model 1**	**model 2**	**model 3**
**Methylation of Chr17:76356190**	**-0.027(-0.043,-0.012)**	**-0.026(-0.042,-0.010)**	**-0.025(-0.040,-0.009)**
**Methylation of Chr17:76356199**	-0.010(-0.020,0.000)	-0.010(-0.019,0.000)	-0.009(-0.019,0.001)

Abbreviation: TCS, triclosan.

model 1: crude model.

model 2: adjusted age, gender, educational level, marital status, average monthly income, smoking status, alcohol status, physical activity, high-fat diet, vegetable and fruit intake, and family history of T2DM.

model 3: model 2 +BMI, PP, TC, TG.

Table S6 Mediation analysis of the association of Ln-TCScrea with T2DM and glucose metabolism-related indicators by methylation level of Chr17:76356190 or Chr17:76356199.

**Mediator**	Total effect	Indirect effect	Direct effect	PE
**Methylation of Chr17:76356190**				
T2DM	**0.124(0.060,0.188)**	**0.018(0.006,0.038)**	**0.109(0.044,0.175)**	**14.54%**
FBG	**0.135(0.072,0.197)**	**0.012(0.004,0.024)**	**0.123(0.061,0.185)**	**8.83%**
INS	**0.157(0.011,0.303)**	0.012(-0.001,0.034)	0.146(-0.001,0.292)	/
HbA1c	**0.067(0.028,0.106)**	0.003(0.000,0.009)	**0.064(0.025,0.103)**	/
Ln-HOMA2-β	**-0.023(-0.038,-0.008)**	**-0.002(-0.005,-0.001)**	**-0.021(-0.035,-0.006)**	**10.00%**
Ln-HOMA2-IR	**0.015(0.004,0.025)**	**0.002(0.001,0.004)**	**0.013(0.002,0.023)**	**11.67%**
**Methylation of Chr17:76356199**				
T2DM	**0.124(0.060,0.188)**	0.009(0.000,0.023)	**0.118(0.053,0.184)**	/
FBG	**0.135(0.072,0.197)**	0.003(-0.000,0.012)	**0.132(0.069,0.194)**	/
INS	**0.157(0.011,0.303)**	0.005(-0.003,0.022)	**0.152(0.006,0.298)**	/
HbA1c	**0.067(0.028,0.106)**	0.002(-0.000,0.006)	**0.065(0.026,0.105)**	/
Ln-HOMA2-β	**-0.023(-0.038,-0.008)**	-0.001(-0.003,0.000)	**-0.022(-0.037,-0.007)**	/
Ln-HOMA2-IR	**0.015(0.004,0.025)**	0.001(0.000,0.002)	**0.014(0.003,0.025)**	/

Abbreviation: TCS, triclosan; T2DM, type 2 diabetes mellitus; FBG, fasting blood glucose; INS, insulin; HbA1c, glycosylated hemoglobin A1c; HOMA2-β, homeostasis model assessment 2 of β cell function; HOMA2-IR, homeostasis model assessment 2 of insulin resistance.

PE* = indirect effect / total effect.

adjusted age, gender, educational level, marital status, average monthly income, smoking status, alcohol status, physical activity, high-fat diet, vegetable and fruit intake, family history of T2DM, BMI, PP, TC, and TG.

Table S7 The results of subgroup analysis.

**characteristics**	***OR* (95%CI)**	***P*-interaction**
	**T2DM**	
**Age, years**		0.858
<55	**1.151(1.015,1.305)**	
55-65	**1.107(1.004,1.221)**	
>65	**1.165(1.015,1.337)**	
**Gender**		0.109
men	**1.198(1.082,1.326)**	
women	**1.091(1.003,1.188)**	
**Educational level**		0.877
never attended school	1.157(0.998,1.342)	
primary school	**1.127(1.001,1.269)**	
junior secondary and above	**1.148(1.039,1.269)**	
**Average monthly income**		0.884
CNY <500	**1.129(1.022,1.248)**	
CNY 500~	**1.137(1.010,1.282)**	
CNY 1000~	**1.160(1.016,1.323)**	
**Physical activity**		0.901
low	**1.145(1.005,1.305)**	
moderate	**1.156(1.042,1.283)**	
high	1.118(0.998,1.253)	
**Smoking status**		0.344
current smoker	**1.257(1.061,1.490)**	
never/past-smoker	**1.121(1.044,1.204)**	
**Alcohol status**		0.720
current drinker	1.153(0.968,1.373)	
never/past-drinker	**1.138(1.060,1.221)**	
**BMI**		0.482
<18.5	/	
18.5-23.9	**1.186(1.073,1.311)**	
>23.9	**1.093(1.007,1.185)**	

Abbreviation: TCS, triclosan; T2DM, type 2 diabetes mellitus.

Table S8 Sensitivity analysis (excluding patients who had diabetes treatment in the last two weeks) (n=784).

**Outcome**	***OR*/ *β* (95%CI)**		
	**model 1**	**model 2**	**model 3**
**T2DM**	1.072(0.999,1.151)	1.073(0.997,1.154)	1.069(0.983,1.163)
**FBG**	**0.074(0.017,0.131)**	**0.073(0.016,0.129)**	**0.059(0.007,0.111)**
**INS**	0.162(-0.004,0.329)	**0.170(0.004,0.3636)**	0.134(-0.022,0.290)
**HbA1c**	0.026(-0.008,0.060)	0.026(-0.008,0.061)	0.019(-0.013,0.050)
**Ln-HOMA2-β**	-0.012(-0.027,0.002)	-0.012(-0.026,0.003)	-0.010(-0.024,0.004)
**Ln-HOMA2-IR**	0.011(-0.001,0.024)	0.011(-0.001,0.024)	0.008(-0.004,0.020)

Abbreviation: TCS, triclosan; T2DM, type 2 diabetes mellitus; FBG, fasting blood glucose; INS, insulin; HbA1c, glycosylated hemoglobin A1c; HOMA2-β, homeostasis model assessment 2 of β cell function; HOMA2-IR, homeostasis model assessment 2 of insulin resistance.

model 1: crude model.

model 2: adjusted age, gender, educational level, marital status, average monthly income, smoking status, alcohol status, physical activity, high-fat diet, vegetable and fruit intake, and family history of T2DM.

model 3: model 2 +BMI, PP, TC, TG.

Table S9 Liquid chromatography gradient elution program

time（min）	0.00	0.50	1.00	3.00	5.00	8.00	8.10	10.00
A pure water（%）	65%	65%	40%	40%	0	0	65%	65%
B methanol（%）	35%	35%	60%	60%	100%	100%	35%	35%

Table S10 HPLC-MS/MS optimized parameters for TCS and TCC.

pollutants	Molecular weight	Retention time (min)	Parent ion (m/z)	Product ion (m/z)	Cone voltage (eV)	Collision energy (eV)
TCS	289.54	5.43	289.02	34.58	20	14
TCC	315.58	6.38	314.93	125.55	10	20

Abbreviation: TCS, triclosan; TCC, triclocarban.

Table S11 Spiked recoveries, coefficients of variation and detection limits of TCS and TCC.

pollutants	Recoveries (%)	Intra−day precision RSD (%)			Inter−day precision RSD (%)			LOD (ng/mL)
		1	5	10	1	5	10	
TCS	126.70	11.13	8.18	7.85	15.42	13.64	12.04	0.0293
TCC	−	15.82	13.64	11.13	15.40	14.72	11.60	0.0066

Abbreviation: LOD, limits of detection; TCS, triclosan; TCC, triclocarban.
